# Droplet Evaporation Process of a Fluorobenzene + n-Octane + Polystyrene Mixture

**DOI:** 10.3390/molecules28155659

**Published:** 2023-07-26

**Authors:** Wei Wang, Zhendong Zhou, Bo Zhou

**Affiliations:** Jiangsu Key Laboratory of Micro and Nano Heat Fluid Flow Technology and Energy Application, School of Environmental Science and Engineering, Suzhou University of Science and Technology, Suzhou 215009, China; wwang2004@dingtalk.com (W.W.); zdzhou2004@dingtalk.com (Z.Z.)

**Keywords:** polymer solution, vapor–liquid equilibrium, activity coefficient models, evaporation process, numerical simulation

## Abstract

The vapor–liquid equilibrium of the fluorobenzene–polystyrene binary polymer solution at 303.15 K was measured using a static pressure device. The vapor–liquid equilibrium of the fluorobenzene–n-octane–polystyrene ternary solution in a partial concentration range under normal pressure was determined using an improved Othmer equilibrium still, in which the octane concentration was low. Three activity coefficient models, poly-NRTL, UNIQUAC, and M-UNIQUAC-LBY, were utilized to correlate the experimental data of binary and ternary solutions, and the component activities of the fluorobenzene–n-octane–polystyrene solution at 303.15 K were predicted. A mathematical model based on the Stefan flow was developed to simulate the evaporation process of composite spherical droplets. The activity predicted by the activity coefficient model was used for numerical simulations, and compared with simulations using the activity following Raoult’s law. The comparative analysis revealed that simulations based on Raoult’s law and activity coefficient models yielded similar results when the mass fraction of fluorobenzene exceeded 0.6. However, in the later stages of evaporation, the calculations based on Raoult’s law predicted a 10% shorter drying time for fluorobenzene. The activity coefficient models provided a better approximation and exhibited similar droplet diameter shrinking behaviors to the actual evaporation process.

## 1. Introduction

**Figure 1:**
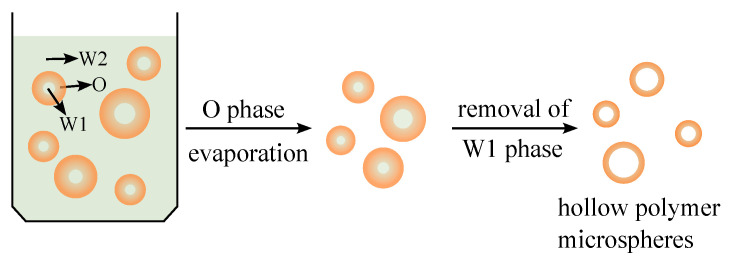
Diagram of composite droplet evaporation.

Polymeric hollow microspheres have been widely applied in various fields, such as biomedicine, chemical catalysis, cosmetics, coatings, and chemical engineering, due to their high specific surface area, low density, and unique cavity structure [1,2]. To prepare polymer microspheres that meet different functional requirements, extensive experimental research has been conducted. Among the preparation methods, the solvent evaporation method is relatively simple and easy to operate, requiring no complex equipment or conditions, making it suitable for the preparation of different types of microspheres [3]. The preparation process involves utilizing the aqueous phase as the inner water phase (W1 phase), an oil-phase solution with the polymeric material as the solute as the middle oil phase (O phase), and a surfactant aqueous solution capable of stabilizing the W1/O composite emulsion as the outer water phase (W2 phase) to obtain W1/O/W2 composite emulsion droplets (Figure 1). The solvent in the O-phase solution is evaporated during the solvent evaporation method, leading to the solidification process, where the concentration and viscosity of the O-phase polymer solution increase. This results in the formation of a high-molecular-weight gel layer encapsulating the inner water phase, yielding polymeric microspheres with an internal cavity. Finally, the removal of the inner water phase through drying produces polymeric hollow microspheres.

The fluorobenzene (FB) polymer system has received significant attention from scholars to satisfy the condition of similar densities between the polymer’s oil and water phases. However, certain issues exist in practical processes, such as non-uniform capsule sizes and difficulties in controlling the evaporation rate of solvents. Chen et al. [4] studied the influence of the FB mass transfer rate on the sphericity and surface smoothness of PAMS products, as well as discussing the mass transfer mechanism. In a subsequent study, Chen et al. [5] introduced hexadecane (C16) into the O phase to reduce the evaporation rate of FB and improve the surface smoothness of the PAMS microspheres. They also investigated the effects of adding octane (C8), dodecane (C12), and hexadecane (C16) to the PAMS system on the curing process [6]. These experiments demonstrated that low-chain-length alkanes prolong the emulsion-curing process by inhibiting the diffusion of the organic solvent from the O phase to the continuous phase. Shao et al. established a mass transfer model for FB inside O/W2 emulsion droplets, describing the mass transfer processes from the droplets to the continuous phase and from the continuous phase to the atmosphere. They developed a numerical solution to the mathematical equation governing the solidification and shrinkage of the droplets based on the finite volume method. Several factors affecting the curing rate and mass transfer process were thoroughly studied, including the number of droplets, initial droplet diameter, initial concentration, and amount of FB added to the continuous phase [7]. In a molecular dynamics simulation conducted by Zhou et al., it was found that polymer molecules have minimal influence on the diffusion of FB molecules in dilute PS/FB solutions. A classical Raoult’s law mass transfer model was used to describe the thermodynamic properties of O/W droplets during the early stages of evaporation in pure water [8]. However, these works lack specific theoretical guidance regarding the influence of various physical parameters during the preparation process of polymeric microspheres. Therefore, experimental research investigating the behavior of polymeric solutions holds importance for understanding the evaporation and solidification of composite droplets.

Phase equilibrium is a critical thermodynamic aspect and forms the theoretical basis for calculating the thermodynamic properties of multi-component systems. Precisely describing the phase equilibrium behavior and thermodynamic properties of polymer solutions is of great significance when studying the physical or chemical processes involved in polymer systems [9]. Several methods are available to determine the solution-phase equilibrium of polymer–solvent compositions in specific systems, such as reverse gas chromatography, piezoelectric sorption apparatus and static pressure method, among others. For example, Saeki et al. [10] employed a piezoelectric adsorption apparatus to measure the activity coefficients of solvents such as benzene, toluene, and chloroform absorbed by polystyrene films at 23.5 ${}^{\circ }C$, while maintaining low solvent mass fractions. Pavlicek et al. [11,12] utilized a microfoamer and employed boiling and static absorption methods to investigate the relationship between the total pressure and composition of the water–polyethylene glycol system. Due to the inherent complexity of solvent–solvent–polymer solutions, the determination of the phase equilibrium becomes more challenging compared to polymer–solvent systems, where the liquid-phase composition can be analyzed solely based on the mass of the absorbed solvent. Funabashi et al. [13] employed a magnetic suspension balance to measure the absorption rate of trace amounts of ethylene by poly(ethylene-methyl acrylate) (EMA)–methyl acrylate (MA) solutions within the temperature and pressure range of 373.28–393.18 K and 5–25 MPa, respectively. In their experiments, a constant mass fraction (10%) EMA/MA solution was used to absorb ethylene, which remained in a molten liquid state under constant temperature and high-pressure conditions. The ethylene in the gas phase was disregarded due to its low mass fraction being absorbed. Pirdashti et al. [14] employed turbidity titration to determine the liquid-phase density and refractive index of ternary solutions, and studied the impact of cation type on the phase equilibrium of polyethylene glycol with formate and sulphate salt aqueous solutions. Santos et al. [15] used a high-pressure phase transition cycling apparatus to determine the gas–liquid- and liquid–liquid-phase equilibria of the ${\mathrm{CO}}_{2}$–methyl methacrylate (MMA)–polydimethylsiloxane (PDMS) system at 27.5 MPa. After adding the two polymers, ${\mathrm{CO}}_{2}$ was injected into the equilibrium chamber using a high-pressure syringe pump, and the system pressure was adjusted using a pressure piston. Finally, the phase behavior of the substances was observed through a light source and a visualization window. Li et al. [16] measured the phase equilibrium of the rubidium chloride–water–polyethylene glycol (PEG) system at temperatures ranging from 288.15 to 308.15 K and correlated the data using different models. They determined the rubidium chloride content in the liquid phase using silver nitrate titration and the PEG content using refractive index calibration, providing a description of the various phase behaviors of the system under atmospheric conditions.

The theory of polymer droplet evaporation and solidification in the presence of interface instability factors has significant implications in optimizing drying processes and investigating the quality of microsphere films. However, there is a lack of ternary oil-phase equilibrium data in the current research. This study focuses on composite droplets composed of conventional fluorobenzene–alkane–polymer ternary oil-phase compositions. The corresponding gas–liquid equilibrium data were measured, and a regression analysis was performed using different activity coefficient models. A mathematical model was also developed to describe the evaporation of spherical composite water droplets (O/W) in air. The model, along with data from different activity coefficient models, was used to numerically solve the evaporation process and evaluate the performance of the different activity models based on the relevant experimental data.

## 2. Results and Discussion

### 2.1. Vapor–Liquid Equilibrium of the Polymer Solution

The experiment used reagents of analytical purity exceeding 99%. The average molecular weight of the PS was 268,000 (measured by gel permeation chromatography). The vapor–liquid equilibrium data for the FB–PS at 303.15 K were measured using the solvent absorption method and the static pressure apparatus designed according to [17]. The vapor–liquid equilibrium data for the FB-octane-PS solution at atmospheric pressure were obtained through an improved Othmer equilibrium still [18]. Instead of a gas chromatograph, a refractometer was used to analyze the composition of the vapor and liquid phases. The experimental setup maintained a temperature stability of 0.1 K, with an average ambient pressure of 101.3 kPa. Similar to other static pressure methods, the experiment in the FB–PS system ignored the variation in the solvent mass fraction inside the vessel before and after reaching equilibrium.

Table 1 and Table 2 present the experimental results. *w* represents the mass fraction of the solvent. Due to the large molecular weight of the polymer, the polymer solution properties are described by the combination of *w* and *a* [19]. Δω represents the error resulting from the initial solvent addition and is calculated as the ratio of the reading error (0.3 mL) to the total solvent volume added. Errors below 0.01 were considered negligible.

### 2.2. Equilibrium Data Regression and Activity Prediction

The binary interaction parameters for the binary and ternary polymer solutions were determined with the experimental data. The estimation process was constrained within the activity calculation range, with the absolute average deviation percentage between the experimental and calculated activity values serving as the error function:

(1) $$AARD\left(\%\right)=\frac{100}{n}\times \sum \left|\frac{a_{i}^{cal}-a_{i}^{exp}}{a_{i}^{exp}}\right|$$

In the above equation, *n* represents the number of experimental values, $a_{i}^{cal}$ represents the predicted values of each model, and $a_{i}^{exp}$ represents the experimental values. The simplex method was employed to regress the vapor–liquid-phase equilibrium data and determine the binary interaction parameters that minimized the error function. The regression analysis resulted in the determination of the binary interaction parameters for each model, along with the corresponding minimum value of the error function as shown in Table 3 and Table 4.

**Figure 2:**
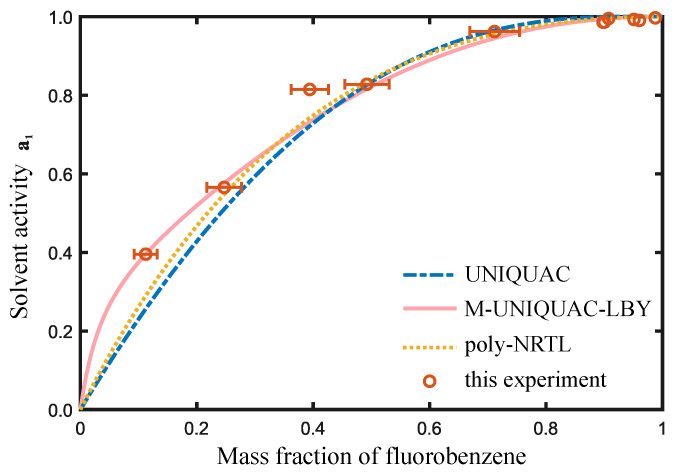
Activity measurement of FB-PS solution at 303.15 K.

**Figure 3:**
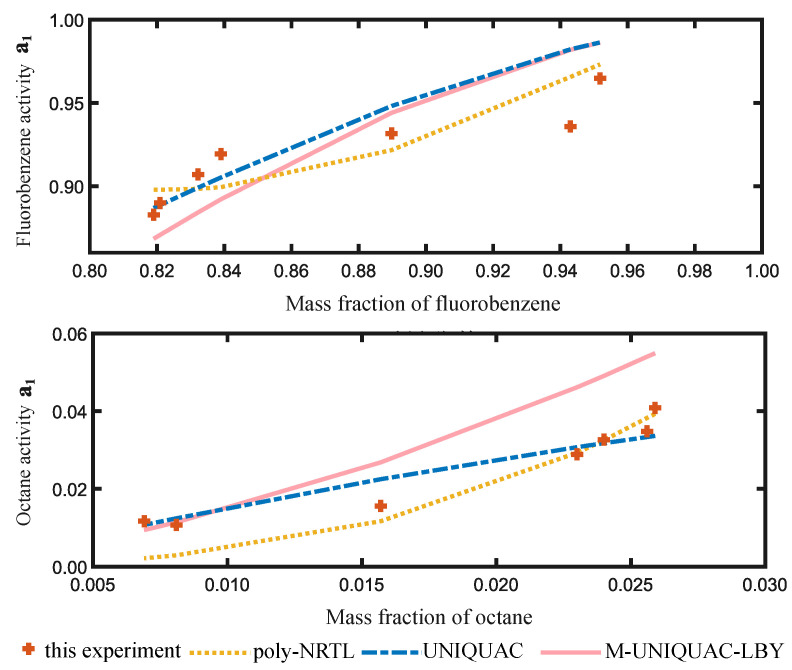
Regression results of activity for FB-octane-PS solution at 303.15 K.

The regression results are shown in Figure 2 and Figure 3, indicating that the poly-NRTL, UNIQUAC, and M-UNIQUAC-LBY [20] models exhibit good adaptability to PS systems. In Figure 2, the UNIQUAC model performs the best for the FB-octane-PS ternary solution. To visually observe the regression effect of each model, we calculated the activity of FB and octane at the same temperature and liquid mass fraction of each model. The *x*-axis of the figure above represents the mass fraction of FB, and the *x*-axis of the figure below represents the mass fraction of octane. In Figure 3, the M-UNIQUAC-LBY model performs the best for the FB–PS binary solution.

**Figure 4:**
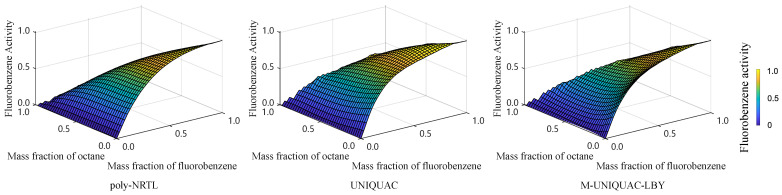
Overview of FB activity in FB-octane-PS solution at 303.15 K.

The predicted activity of the FB-octane-PS solution at 303.15 K is shown in Figure 4. Overall, the ternary solution exhibits positive deviations in the activity coefficient of FB. In calculating the evaporation process of the ternary solution droplet, we applied the activity–concentration relation predicted by all the three types of activity models and compared the corresponding results.

### 2.3. Calculation Results of Ternary Solution Droplet

The simulations were conducted at an environmental temperature of 303.15 K and an atmospheric pressure of 101.3 kPa. The inner water-phase radius of the composite droplet was set to 1 mm ($R_{1}$), while the outer oil-phase radius was set to 2 mm ($R_{0}$). The air boundary was located at a distance of 100 mm ($r_{0}$) away from the droplet center. The initial mass fractions of FB, octane, and PS were set to 0.86, 0.02, and 0.12, respectively. The saturation vapor pressures of FB and octane were obtained from the Antoine equation, and the diffusion coefficients of FB and octane in air were calculated using the Fuller equation [21]. The diffusion coefficients of the components were assumed not to interfere with each other. These simulation parameters were chosen to represent the experimental conditions and enable a comprehensive analysis of the evaporation process.

In the investigation of droplet evaporation, it is commonly assumed that PS, being a non-volatile substance, does not enter the vapor phase. However, the presence of the polymer can cause deviations in solute activity from ideal solution behavior. The previous experiments on the vapor–liquid equilibrium of polymer solutions provide reliable data on the variation of solute activity, eliminating the need for approximate assumptions about solvent activity in previous evaporation models.

**Figure 5:**
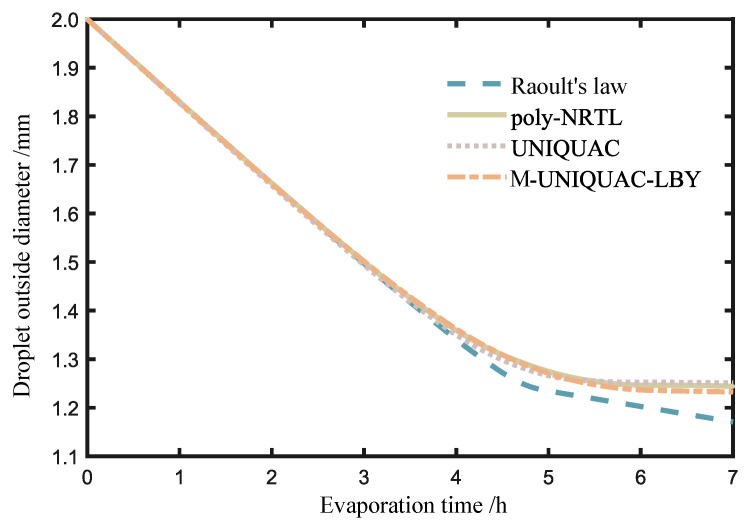
Variation of droplet diameter with evaporation time.

By employing Raoult’s law (activity equals 1) and activity coefficient models, such as poly-NRTL, UNIQUAC, and M-UNIQUAC-LBY, the activities of the components were calculated. A comparison was made between the calculated results for the same system under identical operating conditions as illustrated in Figure 5.

Figure 5 illustrates the variation of the outer diameter of the composite droplet with evaporation time. As the composite droplet evaporates, the volatile components in the oil phase gradually escape, causing the gas–liquid interface of the droplet to shrink inwards. In the figure, the left region represents the early stage of evaporation, during which the results obtained from Raoult’s law and the three activity coefficient models are relatively close. This finding confirms the approximation of using Raoult’s law in previous studies to solve the early-stage evaporation process. However, in the later stages of evaporation, there are noticeable differences between the results obtained from Raoult’s law and the poly-NRTL, UNIQUAC, and M-UNIQUAC-LBY models, and these differences become more pronounced over time.

**Figure 6:**
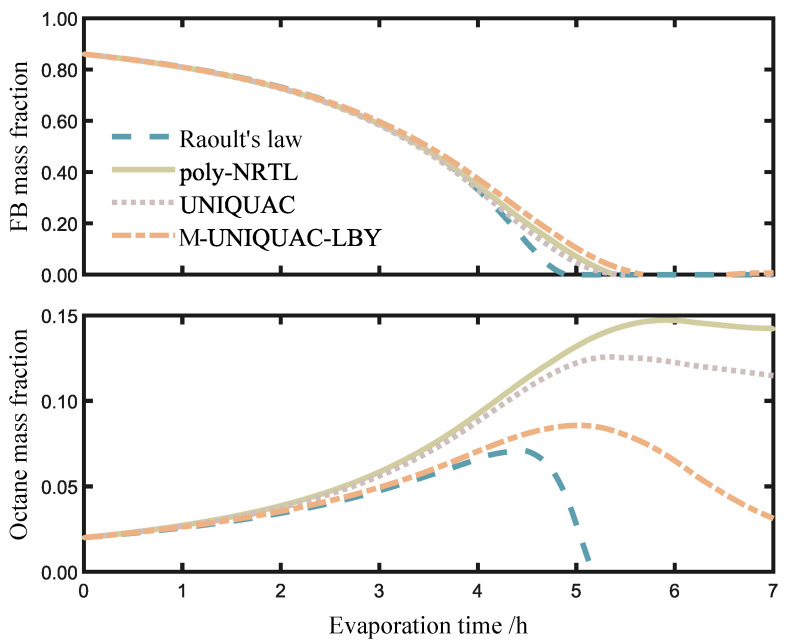
Variation of mass fraction with evaporation time.

Figure 6 compares the variation in the mass fractions of FB and octane during the evaporation process. The upper and lower subplots represent the mass fractions of FB and octane, respectively. Overall, FB exhibits a continuous decrease, while octane initially increases and then decreases. According to our experiment results, as shown in Figure 4, the ternary solution shows positive deviation for the activity coefficient of FB, which means that the repulsive intermolecular forces dominate. Therefore, the two volatile substances do not tend to coexist in the liquid phase, and they escape to the gaseous phase with varying priorities.

During the initial stage of evaporation, which occurs within the time range of 0 to 2.5 h, FB dominates in the oil phase and evaporates at a much faster rate than octane. As a result, the mass fraction of FB decreases relative to octane, while the mass fraction of octane increases.

During the period from 2.5 to 4.5 h, which corresponds to the intermediate stage of evaporation, the mass fraction of FB gradually decreases to a lower level, while the mass fraction of octanes in the liquid phase increases. In the graph, the difference in the mass fraction of FB calculated using Raoult’s law and the activity coefficient models is relatively small, while the mass fraction of octanes shows a significant difference. The main reason for this difference is the different predictions of octane activity by the models at 303.15 K. Additionally, the experimental data obtained for the mass fraction of octanes in the vapor–liquid-phase equilibrium are limited in range and slightly insufficient in quantity. As a result, the models exhibit relatively close predictions for the activity of FB but slightly poorer predictions for octanes. However, overall, the mass fraction of octanes is relatively small during the early and intermediate stages, leading to a minor impact on the calculation of the vapor–liquid interface changes.

During the period from 4.5 h until the end of evaporation, the system enters the late stage of evaporation. At this stage, the mass fraction of FB gradually decreases to 0, while the mass fraction of octanes exhibits an initial increase followed by a gradual decrease in all the models. This is because as FB evaporates, octane gradually becomes the dominant component in the volatile fraction. When FB has evaporated to a certain extent, the mass fraction of octane starts to decrease as octane itself evaporates. Furthermore, in the late stage of evaporation, the change in the droplet outer radius is slow in the poly-NRTL, UNIQUAC, and M-UNIQUAC-LBY models, while in the case of the activity coefficient models, the outer radius predicted by Raoult’s law continues to decrease. In the experimental study conducted by Chen et al. [6] on ternary polymer droplets, it was observed that the actual mass fraction of FB changed relatively slowly over time in this stage, which is consistent with the results obtained from the three activity coefficient models. This is due to the assumption in Raoult’s law that the FB-octane-PS system behaves as an ideal solution, where the evaporation of octane is directly proportional to its mole fraction, deviating from the thermodynamic properties of the polymer solution. On the other hand, in the predictions of activity coefficient models, the evaporation of octane remains at a relatively low level, resulting in a slower change in droplet diameter as the evaporation progresses.

**Figure 7:**
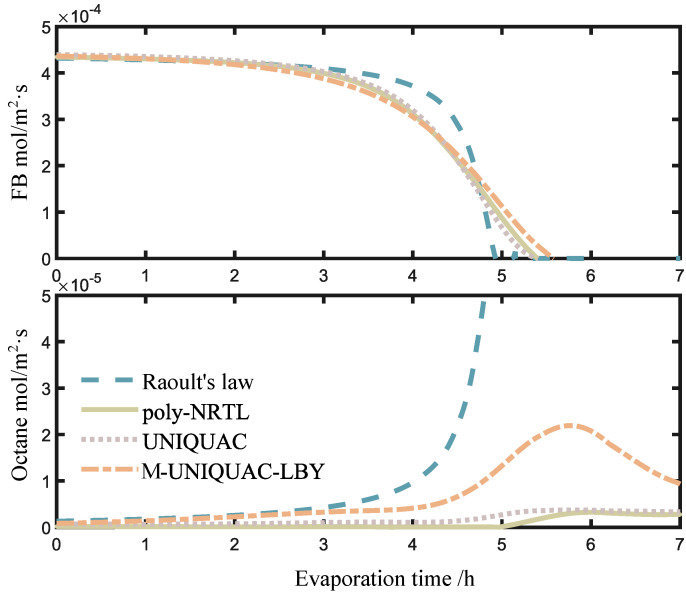
Variation of evaporation rate with time.

In addition, this study compares the evaporation rates of FB and octane as a function of the evaporation time. As shown in Figure 7, the two subplots depict the trends of the evaporation rates for FB and octane over time. In the FB subplot, from 2.5 to 4.5 h, Raoult’s law predicts a faster evaporation rate compared to the activity coefficient models. This is because during this stage, Raoult’s law predicts a higher evaporation rate for FB. Due to the faster evaporation rate predicted by Raoult’s law for FB, the corresponding results enter the late-stage evaporation phase earlier. In the poly-NRTL, UNIQUAC, and M-UNIQUAC-LBY models, the evaporation rates differ numerically due to the different predictions of octane activity. This also suggests the importance of octane-PS equilibrium data for simulating the late-stage evaporation and highlights the need for further investigation into the phase behavior of polymer solutions during this period.

**Figure 8:**
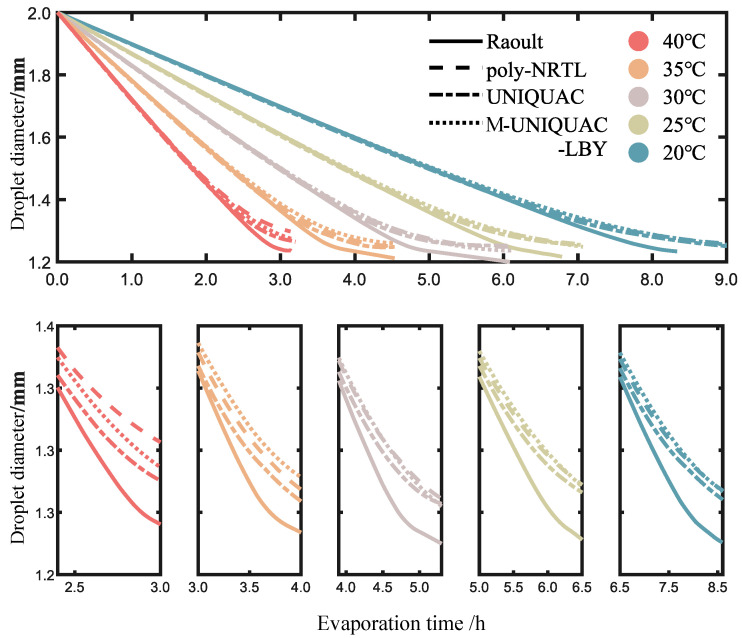
Numerical calculation of evaporation process at different temperatures.

To facilitate comparison with the experimental evaporation and solidification of ternary oil-phase droplets, this study conducted numerical simulations of the evaporation process under different temperature conditions using Raoult’s law and the poly-NRTL, UNIQUAC, and M-UNIQUAC-LBY models. The results are shown in Figure 8. As the temperature increases, the calculated rate of change in droplet diameter by each model gradually increases. It is apparent that in the early stages of evaporation at different temperatures, there is little difference in the numerical results obtained using Raoult’s law and the activity coefficient models. However, in the later stages of evaporation, when the FB concentration decreases to a certain extent, there are some differences between the results obtained using Raoult’s law and the activity coefficient models. In fact, there are also some differences among the poly-NRTL, UNIQUAC, and M-UNIQUAC-LBY models, but these differences are relatively small compared to Raoult’s law. To compare the differences between Raoult’s law and the activity coefficient models, the *x*-axis in the subplots was adjusted to an appropriate range. It can be observed that after adjusting the time scale, the patterns of droplet diameter change at different temperatures are somewhat similar.

## 3. Thermodynamic Model

### 3.1. Vapor–Liquid Phase Equilibrium

Considering the characteristics of the system under investigation, the (φ+γ) method was employed to determine the vapor–liquid-phase equilibrium properties of the polymer solutions. For any vapor–liquid equilibrium system, the fugacities of the vapor and liquid phases are always equal:

(2) $$py_{i}\phi_{i}=f_{i}^{*}\gamma_{i}x_{i}$$

In Equation (2), *p* represents the system pressure; $x_{i}$ and $y_{i}$ represent the molar fraction of component *i* in the vapor and liquid phase, respectively; $\phi_{i}$ represents the coefficient of fugacity of component *i*; $f_{i}^{*}$ represents the fugacity of *i* in the liquid phase; and $\gamma_{i}$ represents the activity coefficient of component *i*.

For the low-pressure phase equilibrium, the vapor phase can be treated as an ideal gas, where $\varphi_{i}$ is equal to 1. The liquid phase should be considered as a non-ideal solution, and is denoted by $\gamma_{i}\neq 1$. Below the critical pressure, the fugacity of liquid at the standard state can be approximated to the saturated vapor pressure of pure components, $p_{i}^{*}$ (assumed sufficiently lower than the total pressure), at the same temperature and pressure. In this case, the phase equilibrium formula simplifies to

(3) $$py_{i}=p_{i}^{*}\gamma_{i}x_{i}$$

(4) $$a_{i}=\frac{p_{i}}{p_{i}^{*}}=\gamma_{i}x_{i}$$

In Equation (4), $a_{i}$ represents the activity of each component. It is generally believed that the boiling point of the polymer is non-existent or very high, and its vapor pressure can naturally be regarded as infinitesimal. Therefore, the polymer is considered to be absent from the vapor phase, and the vapor–liquid equilibrium is primarily focused on the FB and octane. The term $\varphi_{i}^{s}$ can be approximated as the saturation vapor pressure under the same conditions, which can be obtained using the Antoine equation:

(5) lgp*mmHg=A−BBt+Ct+C

The Antoine constants A, B, and C are shown in Table 5 below.

### 3.2. Activity Coefficient Models

The poly-NRTL, UNIQUAC and M-UNIQUAC-LBY models were selected to correlate the experimental data. The first two models are considered classical, and their formulations are well established. In particular, the UNIQUAC model has strong adaptability to multi-component systems, which has been widely concerned by scholars since its inception. There have been quite a few extensions and corrections in the development of this model, and the M-UNIQUAC-LBY model has shown good performance in aqueous solvent and polymer systems [20]. Over the years, the UNIQUAC model has been extended and modified in various ways, demonstrating its excellent performance. In the UNIQUAC-LBY model, Larsen et al. [23] made adjustments to the combining terms and average volume fractions. In the framework of the free volume theory, the volume region in a solution is divided into an intrinsic core volume and a free volume. The volume fraction of a polymer in solution and the distribution of free volume holes are temperature dependent. Building upon this, the M-UNIQUAC-LBY model was optimized, and the expression of activity coefficient was modified as follows:

(6) $$\ln\gamma_{i}=\ln\gamma_{i}^{comb}+\ln\gamma_{i}^{res}+\ln\gamma_{i}^{fv}$$

In Equation (6), $\ln\gamma_{i}^{comb}$, $\ln\gamma_{i}^{res}$ and $\ln\gamma_{i}^{fv}$ are referred to as the combining term, residual term, and free volume term, respectively. In the free volume theory, the volume of matter in a solution consists of two parts, the intrinsic volume and the free volume. In simple terms, the natural volume is the volume occupied by the molecule or atom itself, while the free volume is the volume of the gaps between the natural volumes, which are unequal in size, irregular in distribution, and always unoccupied. Some properties of a polymer, including the polymer glass transition, density fluctuations, molecular diffusion properties, etc., can be well explained by the free volume theory. Some studies have highlighted that the free volume theory has little significance for small molecule systems but is more important in polymer–solvent systems [24].

The residual term in the M-UNIQUAC-LBY model is consistent with the residual contribution in the UNIQUAC model. The combined term is the same as the group contribution in the UNIQUAC-LBY model and can be expressed as follows:

(7) $$\begin{array}{c} \ln\gamma_{i}^{comb}=1-\frac{\xi_{i}}{x_{i}}+\ln\left(\frac{\xi_{i}}{x_{i}}\right)\\ \ln\gamma_{i}^{res}=-q_{i}\ln\left(\sum_{j=1}^{m}\theta_{j}\tau_{ji}\right)+q_{i}-q_{i}\sum_{j=1}^{m}\frac{\theta_{j}\tau_{ij}}{\sum_{k=1}^{m}\theta_{k}\tau_{kj}} \end{array}$$

In contrast to the UNIQUAC model, the new calculation methods are assigned to $\theta_{i}$ and $\xi_{i}$ in Equation (7), representing the average surface area fraction and average volume fraction of molecule *i*, respectively:

(8) θi=zz22qixi∑j=1mzz22qixi

(9) $$\xi_{i}=\frac{x_{i}r_{i}^{\frac{2}{3}}}{\sum_{j=1}^{m}\left(x_{j}r_{j}^{\frac{2}{3}}\right)}$$

**Figure 9:**
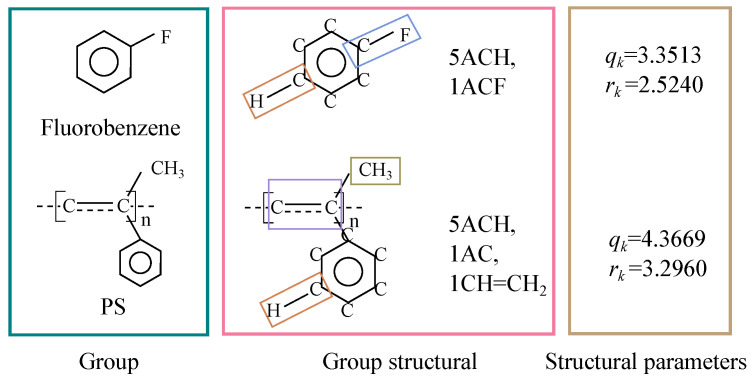
Calculation of group structural parameters [26,27].

In Equations (8) and (9), $\theta_{i}$ and $\xi_{i}$ represent the surface area and volume parameters of molecule *i*, respectively, based on the suggestions of Kikic et al. [25]. They satisfy the relationships $q_{i}=\sum_{k}v_{i}^{\left(k\right)}\left(\frac{z}{2}Q_{k}\right)$ and $r_{i}=\sum_{k}v_{i}^{\left(k\right)}R_{k}$, where $R_{k}$, $Q_{k}$ and the binary interaction parameter τ have the same meanings as in the UNIQUAC model (Figure 9).

Regarding the free volume term,

(10) $$\ln\gamma_{i}^{fv}=\ln\left(\frac{\phi_{i}^{fv}}{\phi_{i}^{h}}\right)+\frac{\phi_{i}^{h}-\phi_{i}^{fv}}{x_{i}}+0.2{\left(1-\frac{\phi_{i}^{fv}}{x_{i}}\right)}^{2}-0.2{\left(1-\frac{\phi_{i}^{h}}{x_{i}}\right)}^{2}$$

In Equation (10), $\phi_{i}^{fv}$ and $\phi_{i}^{h}$ represent the free volume fraction and the core volume fraction of component *i*, respectively:

(11) $$\phi_{i}^{fv}=\frac{x_{i}v_{i}^{fv}}{\sum_{j=1}^{m}x_{j}v_{j}^{fv}}$$

(12) $$\phi_{i}^{h}=\frac{x_{i}v_{i}^{h}}{\sum_{j=1}^{m}x_{j}v_{j}^{h}}$$

where $v_{i}^{fv}$, $v_{i}^{h}$, $v_{i}$ and $v_{i}^{vdw}$ refer to the free volume, core volume, molar volume, and van der Waals volume of component *i*, respectively [28]:

(13) $$\begin{array}{c} v_{i}^{fv}=v_{i}-v_{i}^{h}\\ v_{i}^{h}=1.3\left(v_{i}^{vdw}\right)\\ v_{i}=v_{i}^{vdw}\left(1.3+10^{-3}T\right)\\ v_{i}^{vdw}=15.17\left(r_{i}\right) \end{array}$$

### 3.3. Droplet Evaporation Model Based on Stefan Flow

Reliable vapor–liquid equilibrium data for polymer solutions are of great assistance in numerical simulations of composite emulsion evaporation processes. In this study, we focused on numerically simulating the evaporation process of FB-octane-PS droplets in air.

**Figure 10:**
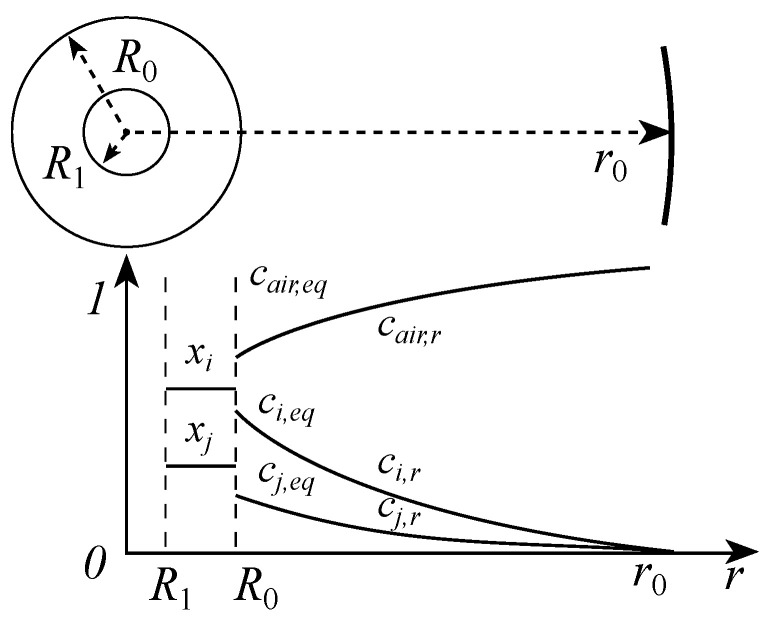
Schematic diagram of the composite droplet evaporation model.

The mathematical model was established as shown in Figure 10, where a spherical droplet containing a water phase is surrounded by an oil phase (comprising FB, octane, and PS) and undergoes evaporation in an ambient air environment. The evaporation environment is assumed to be stable and maintained over a long period, and the droplet evaporation process is considered to be a quasi-equilibrium steady-state process. The following assumptions are made: (1) the temperatures at the air–liquid oil-phase interface, oil-phase interior, oil–water-phase interface, and water-phase interior are constant and equal, and thus only the mass transfer processes are considered; (2) the solubility of air in the liquid phase is negligible, and the alkane and FB vapors in the air can be treated as ideal gases; (3) the droplet is not influenced by gravity, exhibits perfect spherical symmetry, and the self-motion of the oil phase and diffusion within the water phase are neglected; and (4) the droplet is assumed to be sufficiently small, and the concentrations of each component inside the droplet remain uniform. Only the Stefan flow induced by evaporation along the radial direction (R) is considered, taking the droplet center as the origin. Other directional mass transfer processes are neglected in this study.

#### 3.3.1. Convection–Diffusion Equation for the Outer Gas-Phase Environment of Multi-Component Droplets

For the evaporation of multi-component liquid droplets in air, the mole flux of component *i* in the vapor phase, accounting for the Stefan flow, is defined as follows:

(14) $$\begin{array}{c} j_{i}=\upsilon c_{i}\left(r,\tau \right)-D_{i,air}\nabla c_{i}\left(r,\tau \right) \end{array}$$

In Equation (14), $D_{i,air}$ represents the diffusion coefficient of component *i* in air, calculated using the Fuller formula [21]. $c_{i}\left(r,\tau \right)$ represents the molar concentration of component *i* in the vapor phase as a function of diffusion distance *r* and diffusion time τ. υ represents the Stefan flow velocity, determined based on the convective diffusion characteristics according to Stefan’s law:

(15) $$\upsilon =\frac{D_{air}\frac{\partial c_{air}\left(r,\tau \right)}{\partial r}}{c_{air}\left(r,\tau \right)}$$

where $c_{air}$ represents the molar concentration of air, and $D_{air}$ represents the diffusion coefficient of air in the vapor phase. In the vapor–liquid interface, when the fugacity of a component is not much smaller than the ambient pressure, the Stefan flow induced by evaporation always significantly influences the mass transfer intensity of vapor molecules in the vapor phase [29]. In the simulation of the composite liquid droplet evaporation in this study, the initial concentration of FB in the liquid phase is set to be extremely high, leading to a very low concentration of octane in the vapor phase. Therefore, the influence of octane on the diffusion process can be neglected, and $D_{air}$ can be considered equivalent to the diffusion coefficient of FB in air, denoted as $D_{FB,air}$.

The convection–diffusion equation for the vapor phase of multi-component liquid droplets, considering the Stefan flow, can be expressed as follows:

(16) $$\frac{\partial c_{i}\left(r,\tau \right)}{\partial \tau }=D_{i,air}\frac{\partial^{2}c_{i}\left(r,\tau \right)}{\partial r^{2}}-\upsilon \cdot \nabla c_{i}\left(r,\tau \right),\tau >0,R\left(\tau \right)<r<r_{0}$$

The convection–diffusion equation for air $\left(i=air\right)$ also satisfies the above equation. Substituting Equation (15) into it yields

(17) $$\frac{\partial c_{air}\left(r,\tau \right)}{\partial \tau }=D_{air}\frac{\partial^{2}c_{air}\left(r,\tau \right)}{\partial r^{2}}-\frac{D_{air}{\left(\frac{\partial c_{air}\left(r,\tau \right)}{\partial r}\right)}^{2}}{c_{air}\left(r,\tau \right)},\tau >0,R\left(\tau \right)<r<r_{0}$$

#### 3.3.2. Boundary Conditions for the Convection–Diffusion Equation of Multi-Component Droplets in the Outer Region

At the beginning, there are no vapor molecules in the gas phase. Therefore, the initial condition can be written as follows:

(18) $${\left.c_{i}\left(r,\tau \right)\right|}_{\tau =0}=0,R_{0}<r<r_{0}$$

where $r_{0}$ represents the radius at the far end of the gas phase, and $R_{0}$ represents the initial radius of the droplet.

At the far-end boundary of the gas phase, located at coordinate $r_{0}$,

(19) $$\begin{array}{c} {\left.c_{i}\left(r,\tau \right)\right|}_{r=r_{0}}=c_{i,out},\tau >0\\ {\left.c_{air}\left(r,\tau \right)\right|}_{r=r_{0}}=c_{air,out},\tau >0 \end{array}$$

where $c_{air,out}$ represents the concentration of air at $r_{0}$.

Assuming that the solution evaporation maintains in equilibrium at any given time, at the interface of the liquid droplet,

(20) $$\begin{array}{c} {\left.c_{i}\left(r,\tau \right)\right|}_{r=R(\tau )}=c_{i,eq},\tau >0\\ {\left.c_{air}\left(r,\tau \right)\right|}_{r=R(\tau )}=c_{air,eq},\tau >0 \end{array}$$

The equilibrium concentration $c_{i,eq}$ of component *i* at the gas–liquid interface satisfies the gas–liquid equilibrium relationship:

(21) $$c_{i,eq}=\frac{\gamma_{i}p_{i}^{*}x_{i,oil}}{RT}$$

where $x_{i,oil}$ represents the molar fraction of component *i* in the liquid phase, *R* represents the universal gas constant, and *T* represents the evaporation temperature.

We consider the vapor phase to be an ideal gas. Therefore, at any position *r* in the vapor phase, component *i* satisfies the following equation:

(22) $$c_{air}+\sum_{i}c_{i}=\frac{p}{RT}$$

where $c_{i,eq}$ represents the molar concentration of component *i* in the vapor phase when the droplet reaches the equilibrium evaporation state, and *p* represents the total pressure in the vapor phase.

#### 3.3.3. Control Equation for the Radius Variation of Multi-Component Droplets

During the evaporation process of the droplet, the mass conservation law is satisfied. Therefore, neglecting the density variation of the liquid phase, the motion equation for the gas–liquid interface can be expressed as follows:

(23) $$\frac{dR}{d\tau }=\frac{-\sum_{i}M_{i}j_{i}\left|{}_{r=R\left(\tau \right)}\right.}{\rho_{oil}}$$

where $\rho_{oil}$ represents the density of the liquid phase, and $M_{i}$ represents the molar mass of component *i*. The initial condition for this motion differential equation can be expressed as follows:

(24) $$R\left(\tau \right)\left|{}_{\tau =0}\right.=R_{0}$$

#### 3.3.4. Coupled Solution of the Governing Equations

Under the assumption of a quasi-steady state, the convection–diffusion equation for air (17) can be simplified to the following equation:

(25) $$\frac{\partial^{2}c_{air}\left(r,\tau \right)}{\partial r^{2}}-\frac{{\left(\frac{\partial c_{air}\left(r,\tau \right)}{\partial r}\right)}^{2}}{c_{air}\left(r,\tau \right)}=0,\tau >0,R\left(\tau \right)<r<r_{0}$$

By combining the relevant boundary conditions (19) and (20), the analytical solution for the concentration distribution of air, $c_{air}\left(r,\tau \right)$, is given as follows:

(26) $$c_{air}\left(r,\tau \right)=c_{air,out}{\left(\frac{c_{air,eq}}{c_{air,out}}\right)}^{\frac{r_{0}-r}{r_{0}-R\left(\tau \right)}},\tau >0,R\left(\tau \right)\leq r\leq r_{0}$$

By simultaneously considering the steady-state form of Equation (16) and the vapor-phase concentration distribution equation for air (Equation (26)), we can derive the differential equation for the vapor-phase concentration of component *i* as follows:

(27) $$D_{i,air}\frac{\partial^{2}c_{i}\left(r,\tau \right)}{\partial r^{2}}+\frac{D_{air}\ln\left(\frac{c_{air,eq}}{c_{air,out}}\right)}{r_{0}-R\left(\tau \right)}\frac{\partial c_{i}\left(r,\tau \right)}{\partial r}=0,\tau >0,R\left(\tau \right)<r<r_{0}$$

The analytical solution for Equation (27) is as follows:

(28) $$c_{i}=-\frac{D_{i,air}\left(r_{0}-R\left(\tau \right)\right)}{D_{air}\ln\left(\frac{c_{air,eq}}{c_{air,out}}\right)}\times \exp\left(-r\frac{D_{air}\ln\left(\frac{c_{air,eq}}{c_{air,out}}\right)}{D_{i,air}\left(r_{0}-R\left(\tau \right)\right)}+A\right)+B$$

The coefficients A and B in Equation (27) are determined by the boundary conditions in (19) and (20), yielding the following results:

(29) $$A=\ln\left\{\frac{D_{air}}{D_{i,air}}\frac{\left(c_{i,eq}-c_{i,out}\right)\ln\left(\frac{c_{air,eq}}{c_{air,out}}\right)}{\left[1-{\left(\frac{c_{air,eq}}{c_{air,out}}\right)}^{\left(\frac{D_{air}}{D_{i,air}}\right)}\right]\left(r_{0}-R\right)}\right\}+\frac{D_{air}}{D_{i,air}}\frac{\ln\left(\frac{c_{air,eq}}{c_{air,out}}\right)}{r_{0}-R}r_{0}$$

(30) $$B=c_{i,out}+\frac{c_{i,eq}-c_{i,out}}{1-{\left(\frac{c_{air,eq}}{c_{air,out}}\right)}^{\frac{D_{air}}{D_{i,a\mathrm{ir}}}}}$$

The mass fraction of component *i* in the liquid phase can be expressed as follows:

(31) $$Y_{i,oil}=\frac{Y_{i,oil0}\left(R_{0}^{3}-R_{1}^{3}\right)\rho_{oil}-3M_{i}\int_{0}^{\tau }R^{2}j_{i}\left|{}_{r=R\left(\tau \right)}d\tau \right.}{\left(R_{0}^{3}-R_{1}^{3}\right)\rho_{oil}-3\sum_{i}M_{i}\int_{0}^{\tau }R^{2}j_{i}\left|{}_{r=R\left(\tau \right)}d\tau \right.}$$

where $Y_{i,oil}$ represents the mass fraction of component *i* in the liquid phase, and $Y_{i,oil0}$ represents the initial mass fraction of component *i* in the liquid phase. $R_{1}$ represents the internal radius of the droplet, which is assumed to remain constant throughout the evaporation process.

Finally, by simultaneously solving Equations (14), (15), (21)–(23) and (28)–(31), the evaporation rates of each component, the gas–phase concentration distribution, and the variation in droplet diameter with time can be obtained.

## 4. Conclusions

In certain processes, the FB-octane-PS system garners attention due to its density matching with water. This study investigated the vapor–liquid-phase equilibrium characteristics of the FB-octane-PS system. The following conclusions are provided:

(1) Vapor–liquid-phase equilibrium data for FB–PS at 303.15 K and FB-octane-PS at 101.3 kPa, within a specific concentration range, were measured using a static pressure apparatus and an improved Othmer equilibrium still. The obtained data for each system were regressed using the poly-NRTL, UNIQUAC, and M-UNIQUAC-LBY models. The regression results indicated that all three activity coefficient models exhibited strong adaptability to the studied system. Among the models, the UNIQUAC model performed the best, while the poly-NRTL and M-UNIQUAC-LBY models performed slightly less effectively. The activity coefficient models were further utilized to predict the component activities of the FB-octane-PS solution at 303.15 K.

(2) A mathematical model was developed to simulate the evaporation of spherical composite liquid droplets consisting of the oil phase (FB-octane-PS) encapsulating the water phase in the air. Numerical simulations were performed using Raoult’s law, and the three activity coefficient models were used, focusing on the changes in droplet outer diameter, oil-phase composition mass fraction, evaporation rate, and FB vaporization over time. The results showed that there was little difference between Raoult’s law and the activity coefficient models during the early stage of evaporation, but they exhibited some discrepancies in the middle and late stages, with the activity coefficient models being closer to the real process and exhibiting minor differences among themselves. In the evaporation of the oil phase, a gradual decrease in evaporation rate was observed due to the changing vapor pressure of FB, and subsequently, octane started to evaporate. This finding verified previous work. Furthermore, the phase behavior of the octane-PS system played a crucial role in accurately describing the middle and late stages of evaporation for ternary droplets.

## Figures and Tables

**Table 1:** Activity ($a_{i}$) in fluorobenzene-PS at 303.15 K.

*w*	Δω	$a_{i}$
0.987	-	0.997
0.960	-	0.990
0.951	-	0.992
0.907	-	0.995
0.900	-	0.985
0.898	-	0.984
0.711	0.043	0.962
0.492	0.038	0.827
0.394	0.032	0.814
0.247	0.030	0.565
0.112	0.020	0.395

**Table 2:** Temperature and mass fraction in fluorobenzene-octane-PS at atmospheric pressure.

Temperature/°C	Liquid Mass Fraction *w*			Vapor-Phase Mass Fraction *w*		
	Fluorobenzene	Octane	PS	Fluorobenzene	Octane	PS
89.3	0.819	0.026	0.155	0.990	0.010	0.000
89.1	0.821	0.026	0.154	0.987	0.013	0.000
88.5	0.832	0.024	0.144	0.989	0.011	0.000
88.1	0.839	0.023	0.138	0.991	0.009	0.000
87.8	0.890	0.016	0.094	0.997	0.003	0.000
87.7	0.943	0.008	0.049	0.995	0.005	0.000
86.7	0.952	0.007	0.041	0.997	0.003	0.000

**Table 3:** Regression results of fluorobenzene-PS data.

Model	Interaction Parameters	FB-PS		AARD%
Poly-NRTL	$g_{ij}-g_{jj}$	34.3086	17.2779	1.860
UNIQUAC	$\alpha_{ij}$	−174.617	343.576	1.735
M-UNIQUAC-LBY	$\alpha_{ij}$	−250.956	251.201	1.579

**Table 4:** Regression results of fluorobenzene-octane-PS data.

Model	Interaction Parameters	FB-Octane-PS						AARD%
		FB-Octane		Octane-PS		FB-PS		
poly-NRTL	$g_{ij}-g_{jj}$	32.680	43.900	417.540	−916.820	721.100	−383.600	5.778
UNIQUAC	$\alpha_{ij}$	81.818	−0.201	7.354	−205.709	296.137	−338.265	1.508
M-UNIQUAC-LBY	$\alpha_{ij}$	38.909	2.015	155.028	163.241	423.125	−420.969	5.675

**Table 5:** The selection of Antoine coefficients [22].

	A	B	C
FB	6.31155	1381.646	−37.602
octane	6.92374	1355.126	209.517

## Data Availability

Not applicable.

## References

[1] Wei B., Wang S., Song H., Liu H., Li J., Liu N. (2009). A review of recent progress in preparation of hollow polymer microspheres. Pet. Sci..

[2] Yang H.E., Bae Y.C. (2019). Thermodynamic analysis of phase equilibrium and surface tension of ternary polymer solutions. AIChE J..

[3] Li M., Rouaud O., Poncelet D. (2008). Microencapsulation by solvent evaporation: State of the art for process engineering approaches. Int. J. Pharm..

[4] Chen Q., Chen S., Li M., Pan D., Li B., Zhang Z., Qi X. (2018). Influence of fluorobenzene mass transfer on the qualities of poly-*α*-methylstyrene shells. RSC Adv..

[5] Chen Q., Liu M., Pan D., Chen S., Shi R., Qi X., Zhang Z., Li B. (2018). Effects of n-hexadecane on sphericity of poly-*α*-methylstyrene shells. Colloids Surf. A Physicochem. Eng. Asp..

[6] Chen Q., Pan D., Chen S., Liu M., Qi X., Li B. (2019). Resisting effects of alkanes on the stability and deformation of W1-O-W2 droplets. Colloids Surf. A Physicochem. Eng. Asp..

[7] Shao T., Bai L., Yan B., Jin Y., Cheng Y. (2017). Modeling the solidification of O/W-emulsion droplet in solvent evaporation technique. Chem. Eng. Res. Des..

[8] Zhou B., Qi J., Wang W., Xu B., Liu X., Li B., Chen Y. (2022). Influence of oil-phase alkane additives on the evaporation rate of double emulsion curing process. Chem. Eng. Sci..

[9] Marin G.B. (2011). Molecular Thermodynamic Models for Fluids of Chain-Like Molecules, Applications in Phase Equilibria and Micro-Phase Separation in Bulk and at Interface. Advances in Chemical Engineering.

[10] Saeki S., Holste J.C., Bonner D.C. (1981). Sorption of organic vapors by polystyrene. J. Polym. Sci. Part B.

[11] Pavliek J., Bogdani’c G., Wichterle I., Pavel I. (2020). Vapour-liquid equilibria in water + poly(ethylene glycol) systems: New experiments and cumulative thermodynamic processing of all data. J. Chem. Thermodyn..

[12] Pavliek J., Rotrekl J., Bogdani’c G., Wichterle I., Pavel I. (2021). Vapor-liquid and liquid-liquid equilibria in the water + poly(propylene glycol) system. J. Mol. Liq..

[13] Funabashi A., Sato Y., Inomata H. (2018). Measurement and prediction of phase equilibria of ethylene + methyl acrylate + poly(ethylene-co-methyl acrylate) systems. Fluid Phase Equilibria.

[14] Pirdashti M., Heidari Z., Fashami N.A., Arzideh S.M., Khoiroh I. (2021). Phase Equilibria of Aqueous Two-Phase Systems of PEG with Sulfate Salt: Effects of pH, Temperature, Type of Cation, and Polymer Molecular Weight. J. Chem. Eng. Data.

[15] Santos T., Rebelatto E., Chaves B., Lanza M., Oliveira J., Albuquerque E.C., Vieira de Melo S. (2019). High pressure phase equilibrium data for carbon dioxide, methyl methacrylate and poly (dimethylsiloxane) systems. J. Supercrit. Fluids.

[16] Li M., Yu X., Huang Q., Zheng H., Wang L., Feng S., Lai J. (2020). Phase equilibria measurements and correlation of aqueous solvent of PEG4000 with rubidium chloride at (288.15, 298.15, and 308.15) K. J. Chem. Thermodyn..

[17] Liu H., Huang Y., Wang K., Hu Y. (2002). Vapor-liquid equilibria for mixed solvents-polymer systems, measurement and correlation. Fluid Phase Equilibria.

[18] Li S., Huang X., Huang Q., Guo T., Yun S., Ban C., Shen G. (2018). Vapor-liquid equilibrium in the binary and ternary systems containing ethyl propionate, ethanol and alkane at 101.3 kPa. Fluid Phase Equilibria.

[19] Kang C.H., Sandler S.I. (1987). Phase behavior of aqueous two-polymer systems. Fluid Phase Equilibria.

[20] Amirsoleymani A., Bakhshi H., Shabanian S.R., Movagharnejad K. (2020). Phase equilibria of binary and ternary polymer solutions using modified UNIQUAC-based local composition model. J. Therm. Anal. Calorim..

[21] Carey V.P. (1988). The properties of gases & liquids: 4th Edition. Robert C. Reid, John M. Prausnitz, and Bruce E. Poling, McGraw-Hill Book Company, New York, NY, 1987, 741 pages, $49.50. Exp. Therm. Fluid Sci..

[22] Haynes W.M. (1990). CRC Handbook of Chemistry and Physics.

[23] Larsen B., Rasmussen P., Fredenslund A. (1987). A modified UNIFAC group-contribution model for prediction of phase equilibria and heats of mixing. Ind. Eng. Chem. Res..

[24] Oishi T., Prausnitz J.M. (1978). Estimation of Solvent Activities in Polymer Solutions Using a Group-Contribution Method. Ind. Eng. Chem. Process Des. Dev..

[25] Kikic I., Alessi P., Rasmussen P., Fredenslund A. (1980). On the combinatorial part of the UNIFAC and UNIQUAC models. Can. J. Chem. Eng..

[26] Gmehling J., Kolbe B., Jorgensen S.S., Rasmussen P. (1979). Vapor-Liquid Equilibria by UNIFAC Group Contribution, Revision and Extension 2. 1979. Ind. Eng. Chem. Process Des. Dev..

[27] Brelvi S.W. (1982). Simple correlations for UNIQUAC structure parameters. Ind. Eng. Chem. Process Des. Dev..

[28] Krevelen V. (2009). Properties of Polymers: Their Correlation with Chemical Structure; Their Numerical Estimation and Prediction from Additive Group Contributions.

[29] Vlasov V.A. (2020). Diffusion-kinetic model of liquid evaporation from a Stefan tube: A solution to the Stefan diffusion problem. Int. J. Heat Mass Transf..

